# 60941 Vaginal pH predicts cervical intraepithelial neoplasia-2 regression in women living with human immunodeficiency virus

**DOI:** 10.1017/cts.2021.465

**Published:** 2021-03-30

**Authors:** Katherine Michel, Christine Colie, Robert D. Burk, L. Stewart Massad, Cuiwei Wang, Michaeline Hebron, Charbel Moussa, Gypsyamber D’Souza, Lisa Rahangdale, Lisa Flowers, Joel Milam, Joel M. Palefsky, Howard Minkoff, Howard D. Strickler, Seble G. Kassaye

**Affiliations:** 1 Georgetown University; 2 Albert Einstein College of Medicine; 3 Washington University in St. Louis; 4 Johns Hopkins University; 5 University of North Carolina School of Medicine; 6 Emory University; 7 University of California Irvine; 8 University of California San Francisco; 9 Maimonides Medical Center

## Abstract

ABSTRACT IMPACT: The potential to use vaginal pH as a low cost, non-invasive diagnostic test at the point of CIN2 diagnosis to predict worsening of cervical disease. OBJECTIVES/GOALS: We previously reported that persistence/progression of cervical intraepithelial neoplasia-2 (CIN2) was uncommon in women living with HIV (WLH) from the Women’s Interagency HIV Study (WIHS, now MWCCS). Here we examined additional factors that may influence CIN2 natural history. METHODS/STUDY POPULATION: A total of 337 samples from 94 WLH with a confirmed CIN2 diagnosis were obtained from the MWCCS. 42 cervicovaginal HPV types and 34 cervicovaginal cytokines/chemokines were measured at CIN2 diagnosis (94 samples) and 6-12 months prior to CIN2 diagnosis (79 samples). Covariates, including CD4 count and vaginal pH, were abstracted from core MWCCS visits. Logistic regression models were used to explore CIN2 regression (CIN1, normal) vs. persistence/progression (CIN2, CIN3). Log rank tests, Kaplan Meier method, and Cox regression modeling were used to determine CIN2 regression rates. RESULTS/ANTICIPATED RESULTS: The most prevalent HPV types were HPV54 (21.6%) and 53 (21.3%). 33 women (35.1%) had a subsequent CIN2/CIN3 diagnosis (median 12.5 years follow-up). Each additional hr-HPV type detected at the pre-CIN2 visit associated with increased odds of CIN2 persistence/progression (OR 2.27, 95% CI 1.15, 4.50). Higher vaginal pH (aOR 2.27, 95% CI 1.15, 4.50) and bacterial vaginosis (aOR 5.08, 95% CI 1.30, 19.94) at the CIN2 diagnosis visit associated with higher odds of CIN2 persistence/progression. Vaginal pH >4.5 at CIN2 diagnosis also associated with unadjusted time to CIN2 persistence/progression (log rank p=0.002) and a higher rate of CIN2 persistence/progression (adjusted hazard ratio [aHR] 3.37, 95% CI 1.26, 8.99). Cervicovaginal cytokine/chemokine levels were not associated with CIN2 persistence/progression. DISCUSSION/SIGNIFICANCE OF FINDINGS: We found relatively low prevalence of HPV16/18 in this cohort. Elevated vaginal pH at the time of CIN2 diagnosis may be a useful indicator of CIN2 persistence/progression and the rate of persistence/progression.

